# Accelerated Orthodontics: Getting Ahead of Ourselves With Corticotomy

**DOI:** 10.7759/cureus.50858

**Published:** 2023-12-20

**Authors:** Dhwani Suchak, Ranjit Kamble, Nishu Agarwal, Unnati Shirbhate, Renuka Talla

**Affiliations:** 1 Orthodontics, Sharad Pawar Dental College, Datta Meghe Institute of Higher Education and Research, Wardha, IND; 2 Periodontics, Sharad Pawar Dental College, Datta Meghe Institute of Higher Education and Research, Wardha, IND

**Keywords:** alveolar bone remodelling, rapid orthodontics, adult treatment, corticotomy, tooth movement

## Abstract

Corticotomy-assisted orthodontics is an emerging technique that combines orthodontic treatment with selective alveolar corticotomy to accelerate tooth movement and optimize treatment outcomes. This case report presents the successful application of corticotomy-assisted orthodontics in the management of a complex malocclusion. Following a comprehensive orthodontic assessment and treatment planning, corticotomy-assisted orthodontics was selected as the treatment approach. The surgical procedure involved selective alveolar corticotomy in the affected regions, followed by initiation of orthodontic mechanics. The case was closely monitored at regular intervals, and treatment progress was assessed using clinical examination and radiographs. The combined approach led to significantly accelerated tooth movement, a reduction in treatment time, and improved patient satisfaction. This case report underscores the potential benefits of corticotomy-assisted orthodontics in complex cases, providing clinicians with valuable insights into its successful application and the considerations involved in treatment planning and execution. Further research and long-term follow-up are warranted to elucidate the long-term stability and outcomes of this approach.

## Introduction

Adult patients are increasingly requesting fixed orthodontic appliances. Adult and juvenile orthodontic treatment have significant psychological, biological, and therapeutic differences. Orthodontists are driven to research potential orthodontic tooth movement procedures. Adults have almost no growth left of the skeletal bases compared to children, and hyalinization is becoming more likely with treatment. Furthermore, cell mobilization and collagen fiber conversion in adults are much slower than in children. Finally, because teeth are contained in rigid alveolar bone, patients with increasing age are more likely to develop periodontal problems [1].

Adults' periodontal health requires extra attention because they are more prone to have suffered periodontal disease in the past. Although the global frequency of gingival inflammation is substantial, epidemiologic studies suggest that chronic periodontal disease impacts only a small fraction of people worldwide, ranging from 8% to 30%. Periodontal disease is more common in patients with a history of the disease, and it is more common among specific teeth, particularly the upper and lower molars, because the anatomy of the molars makes them susceptible to plaque accumulation [2].

These characteristics differentiate adult orthodontic therapy and present unique challenges, calling for particular concepts and techniques like the use of clear appliances, reduced treatment times, minimal forces, and more accurate tooth movements [3].

The amount of force applied and the biological responses of the periodontal ligament can be used to modulate orthodontic movement. Because of changes in blood flow, this orthodontic stress would cause inflammation around the periodontal ligament (PDL), leading to the release of agents of inflammation such as colony-stimulating factors, growth factors, arachidonic acid metabolites, cytokines, and neurotransmitters. These secretions include macrophage colony-stimulating factor (M-CSF), receptor activator of nuclear factor kappa B ligand (RANKL), and osteoprotegerin (OPG) by osteoblasts, all of which are important in tooth movement. The ligand attaches to its receptor, the receptor activator of nuclear factor kappa B (RANK), which is present on top of osteoclast cells during the embryonic phase. Ligand/receptor communication is required for osteoclast activity, differentiation, and survival. The orthodontic treatment can last anywhere from 24 to 36 months, increasing the chances of complications, such as resorption of roots externally, periodontal issues, and patient willingness. Orthodontists are driven to research the potential to bring about advances in orthodontic tooth movement procedures [4].

The introduction of corticotomy-assisted orthodontic treatment (CAOT) opened up new avenues and provided answers to several constraints in adult orthodontic treatment [3]. 

Since the 1800s, surgically aided orthodontic tooth movement has been used. Bryan described corticotomy-assisted tooth movement for the first time in 1893 [1]. However, Kole was the first to use it as a method of supra apical horizontal osteotomy in 1959 [5]. By replacing the horizontal osteotomy cut beyond the tooth apices with a subapical horizontal corticotomy cut, Suya et al.(1991) modified Kole's approach [6]. The accelerated osteogenic orthodontics technique (AOO) was developed by Wilcko et al., which in recent times is also known as periodontally accelerated osteogenic orthodontics (PAOO; 2000, 2001, 2003, 2008) that combined corticotomy surgery with alveolar grafting [7-10]. Full-mouth fixed orthodontic equipment, as well as complete mucoperiosteal flaps and labial and lingual corticotomies around teeth to be relocated, was required for this method. The raised flap was approximated back in place after a bone graft of demineralized freeze-dried bone and bovine bone, which has shown new bone formation without any severe complications, and clindamycin phosphate solution (10 mg/ml) was mixed with the bone graft just before placing it on the site [11]. The orthodontic appliance was activated two weeks after the surgery to begin tooth movement and then every two weeks thereafter [12].

## Case presentation

**Figure 1:**
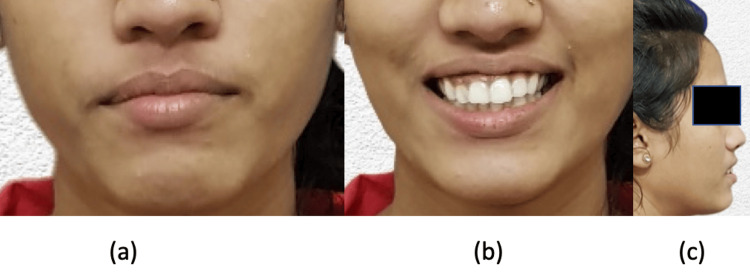
Pre-treatment extraoral images: (a) frontal, (b) smiling, (c) profile

**Figure 2:**
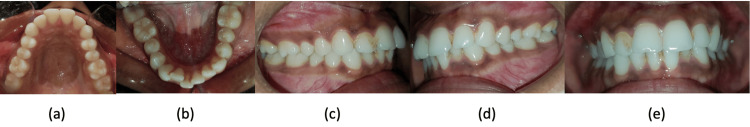
Pre-treatment intraoral images: (a) maxillary arch, (b) mandibular arch, (c) right molar in occlusion, (d) left molar in occlusion, (e) anterior in occlusion

**Figure 3:**
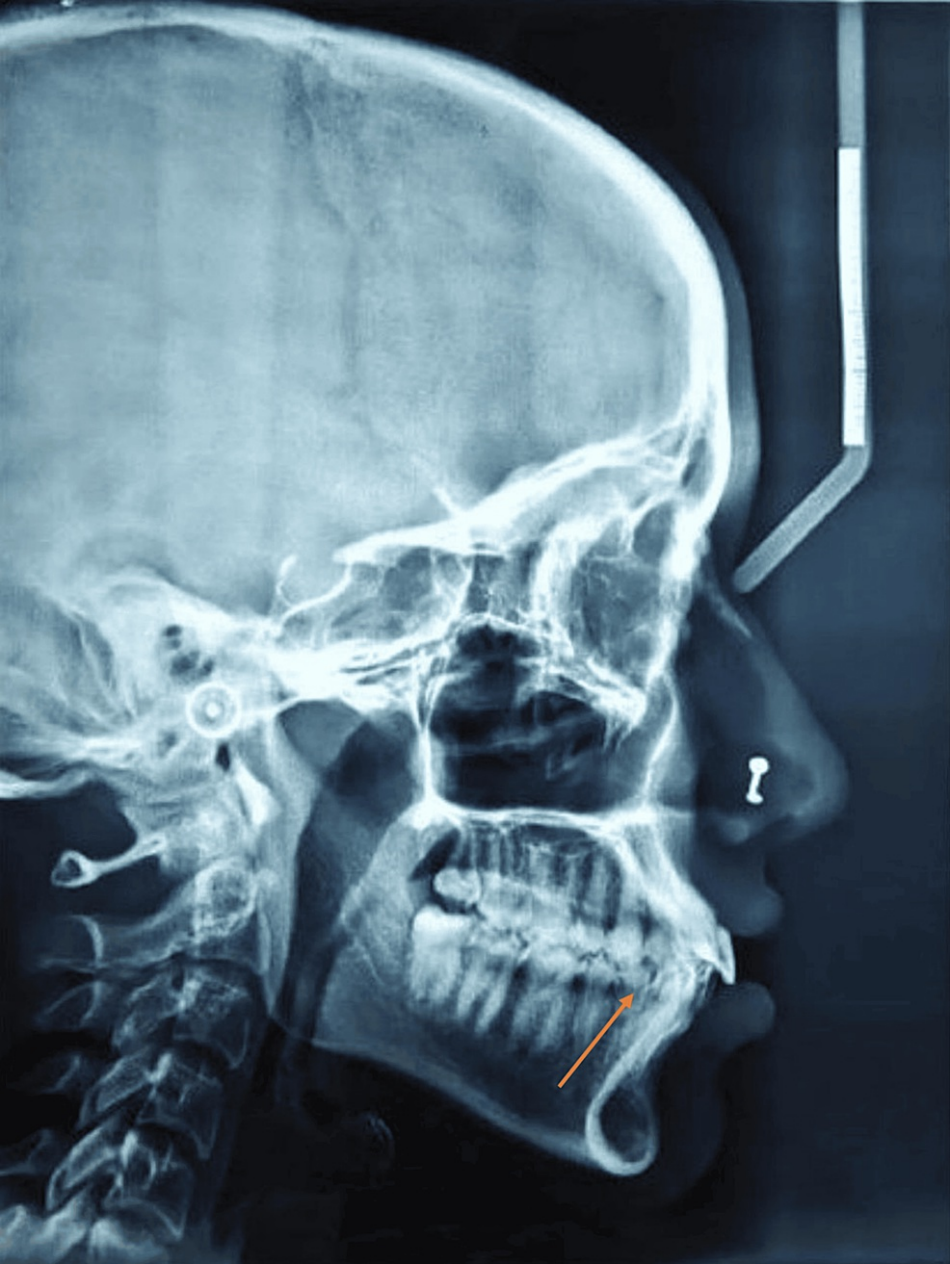
Pre-treatment lateral cephalogram

**Figure 4:**
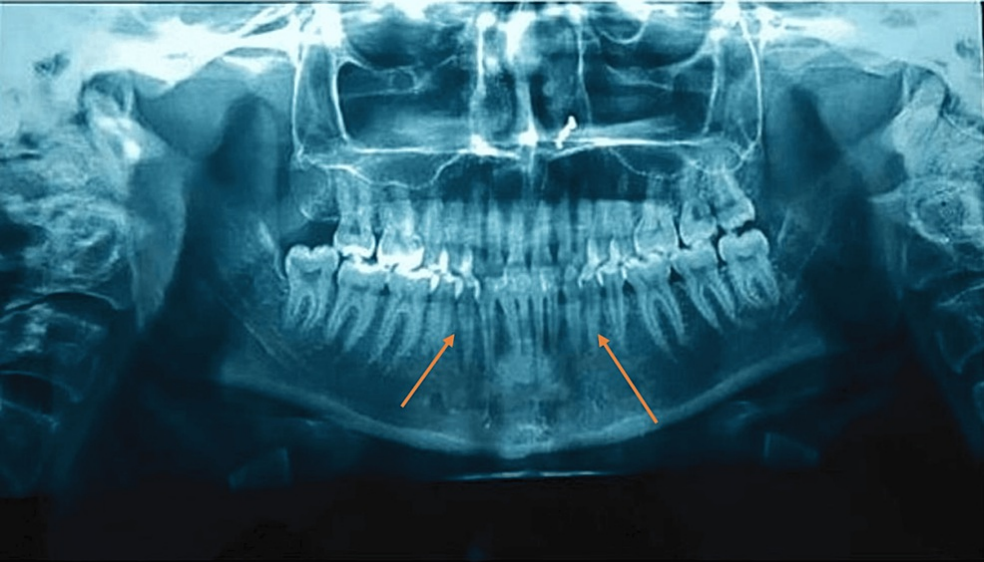
Pre-treatment orthopantomogram

A 19-year-old female patient visited the department with the primary complaint of malaligned teeth. The pre-treatment extraoral photos (Figure 1) revealed a straight profile with an acute nasolabial angle. The intraoral photographs (Figure 2) revealed Angle's class I molar and class I canine relation on both sides, constricted maxillary, and crowded mandibular arches. The pre-treatment lateral cephalogram showed skeletal class II with proclination of upper and lower incisors (Figure 3 and Table 1). The pre-treatment orthopantamogram showed missing 18 and Angleis class I molar relation (Figure 4). The problem list for this patient has been mentioned in Table 2.

**Table 1:** Pre-treatment lateral cephalogram values SNA: Sella-nasion to point A angle; SNB: Sella-nasion to point B angle; ANB: angle between point N to A and point N to B; IMPA: incisor mandibular plane angle; NA - nasion to point A; NB - nasion to point B; OP - occlusal plane

Measurement	Mean value	Pre-treatment
SNA (°)	82	82
SNB (°)	80	79
Mandibular plane angle (°)	21.9	17
Effective maxillary length (mm)	92.7 ± 2.3	97
ANB (°)	2	3
Beta angle (°)	27 - 33	23
A-B (ll to OP) (mm)	-0.4 ± 2	7
1 to NA (°)	22	32
1 to NA (mm)	4	7
1 to NB (°)	25	28
1 to NB (mm)	4	6
IMPA (°)	90	100
Effective mandibular length (mm)	120 ± 3.4	97
Nasolabial angle (°)	102 ± 4	90
Saddle angle (°)	123 ± 5	119

**Table 2:** Problems list

Dental	Skeletal	Soft tissue
Proclination of upper and lower incisors	Class II skeletal bases	Acute nasolabial angle
Crowding in lower incisors		Potentially competent lips
Increased overjet		

Treatment objectives and plan

The treatment objectives for the following case were to correct proclined maxillary and mandibular incisors, correct crowding of lower anteriors, and correct overjet.

Since the patient desired a shorter treatment duration, the treatment plan was formulated according to the needs of the patient (Table 3).

**Table 3:** Treatment plan MBT - Mclaughlin, Bennett, Trevisi system

Orthodontic plan	Surgical plan
Case was started with type A anchorage preparation by banding all second molars and 0.022” MBT slot prescription	Corticotomy to accelerate tooth movement
Extraction of 14, 24, 34, 44	
Initial leveling and alignment	
Closure of extraction spaces	
Finishing and detailing	
Retention	

Treatment progress

MBT 0.022" (Fox series, Libral, India) slot prescription was used for this case. Extraction of 14, 24, 34, 44 was carried out. Initial leveling and alignment were carried out with 0.016'', 0.016x0.022'', 0.017x0.025'' nickel-titanium (NiTi), and finally, 0.017x0.025'' stainless steel (SS) archwires. The pre-operative orthodontic phase required four months. Following leveling and alignment, the patient was scheduled for corticotomy surgery.

For the corticotomy surgery, 1% lignocaine with adrenaline was delivered. At the extraction location, a supraperiosteal vertical incision was made. To elevate the periosteum, a periosteal elevator was used, and a tunnel was made to reveal the buccal cortical bone, and it was extended apically to the region of corticotomy. A Mectron piezoelectric apparatus (Mectron, Italy) with continuous irrigation was used to perform a mild corticotomy to minimize injury and necrosis of the surrounding tissues during and after surgery.

**Figure 5:**
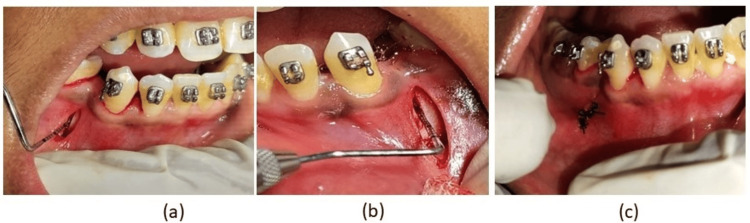
Surgical steps

The bone was cut vertically and somewhat superficially to expose the bone marrow. After the procedure, simple interrupted sutures were placed over the incision. The sutures were removed after five to seven days (Figure 5).

**Figure 6:**
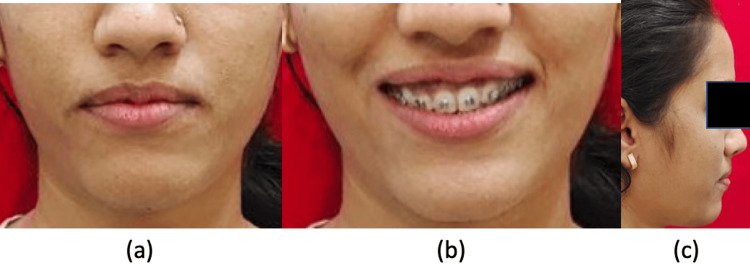
Extraoral images showing the progression of treatment: (a) frontal, (b) smiling, (c) profile

**Figure 7:**
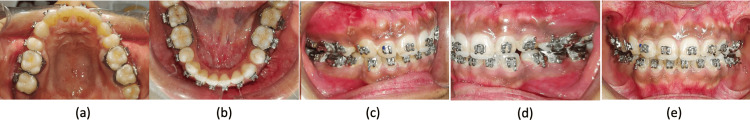
Intraoral images showing space closure: (a) maxillary arch, (b) mandibular arch, (c) right molar in occlusion, (d) left molar in occlusion, (e) anterior in occlusion

**Figure 8:**
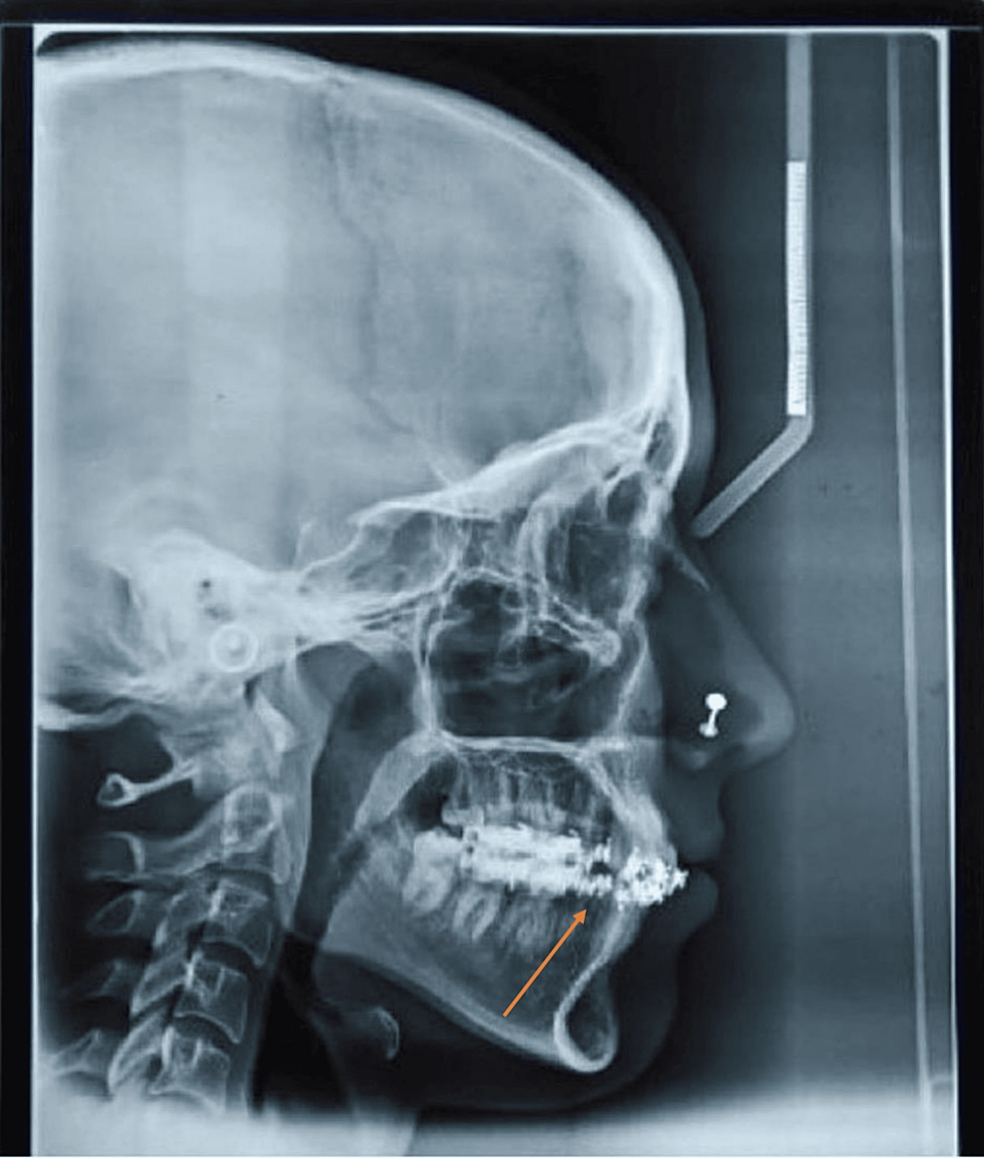
Post-treatment lateral cephalogram

**Figure 9:**
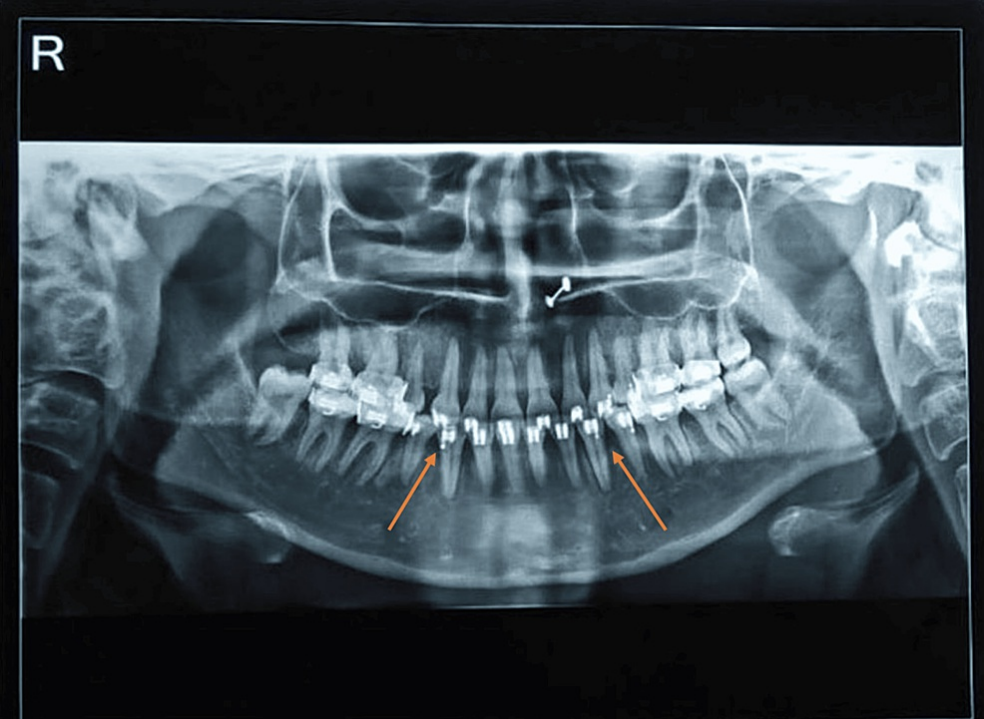
Post-treatment orthopantomogram

**Figure 10:**
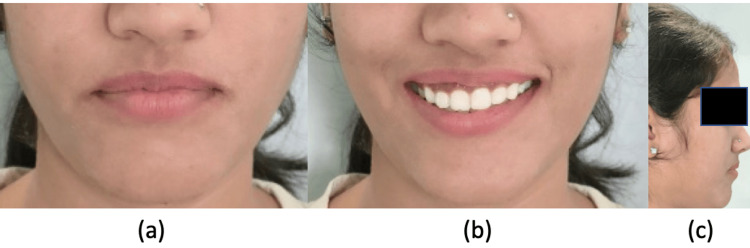
Post-treatment extraoral images: (a) frontal, (b) smiling, (c) profile

**Figure 11:**
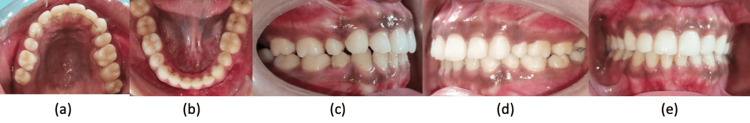
Post-treatment intraoral images: (a) maxillary arch, (b) mandibular arch, (c) right molar in occlusion, (d) left molar in occlusion, (e) anterior in occlusion

After corticotomy surgery, a healing phase of 14 days was given, following which 0.019x0.025” SS archwires with E-chains with an activation period of 15 days. The duration for the entire space closure was four months (Figures 6, 7). After complete retraction, the patient's primary complaint of malaligned teeth was corrected. Patient satisfaction with an accelerated treatment was also fulfilled at the end of the treatment. An improved facial profile along with competency of lips were appreciated. Post-treatment cephalometric corrections were done in the lateral cephalogram and orthopantomogram (OPG) that were recorded just before debonding (Figures 8, 9). The pre- and post-treatment lateral cephalogram values are compared in Table 4. The treatment was completed in 10 months. Bonded lingual permanent retainers were given for a year (Figures 10, 11).

**Table 4:** Comparison of pre-treatment and post-treatment lateral cephalogram values SNA: Sella-nasion to point A angle; SNB: Sella-nasion to point B angle; ANB: angle between point N to A and point N to B; IMPA: incisor mandibular plane angle; NA - nasion to point A; NB - nasion to point B

Measurement	Pre-treatment	Post-treatment
SNA (°)	82	83
SNB (°)	79	80
Mandibular plane angle (°)	17	18
Effective maxillary length (mm)	97	97
ANB (°)	3	3
Beta angle (°)	23	23
A-B (ll to OP)(mm)	7	4
1 to NA (°)	32	26
1 to NA (mm)	7	5
1 to NB (°)	28	26
1 to NB (mm)	6	4
IMPA (°)	100	93
Effective mandibular length(mm)	97	97
Nasolabial angle (°)	90	107
Saddle angle (°)	119	119

## Discussion

Oral surgical treatments, such as corticotomy, can alter bone biology, hastening tooth mobility and shortening treatment time. According to Gil et al., corticotomy-facilitated orthodontic treatments required a reduced treatment length of 8.85 months compared to conventional orthodontic therapy, which lasted an average of 16.4 months [13]. Corticotomy methods are based on a complex physiologic mechanism called the regional acceleratory phenomenon (RAP), which involves an enhanced rate of bone turnover [8]. RAP promotes rapid orthodontic tooth movement by temporarily increasing localized tissue remodeling, the initial phase of which is characterized by enhanced osteoclastic activity leading to cortical bone porosity. It has been noted that the regional acceleratory phenomenon starts shortly after surgery, peaking around one to two months and can extend up to six to 24 months [14].

Another fundamental concept of corticotomy is that it decorticates the bone, causing transient osteopenia and a brief drop in mineral content. Within 20-55 days, the osteogenic cells begin laying down copious calcium deposits, followed by bone mineralization. Orthodontic brackets assist in shifting teeth more quickly during this transient period because the juvenile bone (woven bone) provides less resistance to orthodontic forces as compared to the cortical bone [15,16]. Baloul et al. proposed that alveolar decortication hastens the migration of teeth during the initial stages, resulting in bone resorption and formation during the initial phases of therapy [15].

According to Kim et al., their experiment on cats revealed that concision might be an effective technique for speeding orthodontic tooth movement while also remodeling the alveolar bone [16]. All of these findings were clinically translated in the present case by the shorter time necessary for orthodontic treatment. PAOO also allows for greater orthodontic tooth movement without unnecessary problems, giving it a superior treatment option in certain patients that would otherwise necessitate orthognathic surgery [10,16].

## Conclusions

In summary, accelerated orthodontics using corticotomy represents a paradigm shift in orthodontic treatment, offering a viable alternative for those seeking expedited results. While challenges and considerations exist, the potential benefits make it an exciting area for continued exploration and integration into contemporary orthodontic practice. The corticotomy-assisted technique accelerated tooth movement significantly. The rate of tooth movement was two to four times greater than that of conventional orthodontics, which usually takes about 16 months.
